# Single OnabotulinumtoxinA Session Add-On to Carbamazepine or Oxcarbazepine in Treatment-Refractory Trigeminal Neuralgia: A Case Series with 24-Week Follow Up

**DOI:** 10.3390/toxins15090539

**Published:** 2023-08-31

**Authors:** Georgia Xiromerisiou, Ioannis C. Lampropoulos, Emmanouil V. Dermitzakis, Michail Vikelis, Chrysoula Marogianni, Dimitrios Mysiris, Andreas A. Argyriou

**Affiliations:** 1 Department of Neurology, Faculty of Medicine, School of Health Sciences, University Hospital of Larissa, 41110 Larissa, Greece; 2 Respiratory Medicine Department, Faculty of Medicine, University of Thessaly, 41110 Larissa, Greece; i.ch.lampropoulos@gmail.com; 3 Euromedica General Clinic, 54645 Thessaloniki, Greece; manolis.dermitzakis@gmail.com; 4 Headache Clinic, Mediterraneo Hospital, 16675 Glyfada, Greece; mvikelis@headaches.gr; 5 Faculty of Medicine, School of Health Sciences, University Hospital of Larissa, 41110 Larissa, Greece; c.marogianni@gmail.com (C.M.); dim_mysiris@hotmail.com (D.M.); 6 Headache Outpatient Clinic, Department of Neurology, Agios Andreas State General Hospital of Patras, 26335 Patras, Greece; andargyriou@yahoo.gr

**Keywords:** neuropathic pain, treatment-refractory trigeminal neuralgia, onabotulinumtoxinA, symptomatic therapy

## Abstract

We sought to assess the efficacy of combining onabotulinumtoxinA (BoNTA) as add-on therapy to carbamazepine or oxcarbazepine in treatment-refractory patients with trigeminal neuralgia (TGN) who failed to respond (less than 30% response rate) to adequate monotherapy. We conducted a retrospective study on 15 patients with a definite diagnosis of TGN, according to the established criteria, and underwent BoNTA as part of their treatment plan. A single BoNTA session was administered subcutaneously, according to patients’ perceived zone of pain, at different dosages ranging from 30 to 200 units (mean ± standard deviation: 87.3 ± 39.2). All patients (15/15; 100%) reported large reductions in the severity of their TGN-related neuropathic pain. The mean pain score on the VAS scale significantly decreased from 9.3 ± 1.1 to 3.7 ± 1.2 at 2 weeks after injecting BoNTA (*p* < 0.001) and remained stable at 4 and 24 weeks post-injection. Regarding the impact of BoNTA on patients’ health-related quality of life, there were significant improvements in both the physical and mental health domains (*p* < 0.05) of SF-36 tool. BoNTA may be a safe and effective treatment option for patients with refractory TGN when added on to carbamazepine or oxcarbazepine. The use of a single BoNTA session for TGN treatment may be an alternative to surgical interventions and as add-on treatment to oral medications, providing patients with a minimally invasive, effective, safe and well-tolerated option.

## 1. Introduction

Neuropathic pain is defined as a pain of nervous origin which is initiated or caused by a primary lesion or dysfunction of the peripheral or central nervous system [1]. The three main entities of central-origin craniofacial neuropathic pain are trigeminal neuralgia (TGN), postherpetic neuralgia and migraine, while on clinical grounds, TGN can be perceived as one of the most painful and common types of neuropathic pain generated within the central nervous system (CNS) [2]. In addition, TGN is also commonly seen, as epidemiological studies have revealed that approximately 4 to 28.9/100,000 people worldwide suffer from TGN. Patients with advanced age (>60 years) and females over males are mostly affected [3]. It involves the fifth cranial nerve, and pain is usually described as paroxysmal, intense, stabbing, reminiscent of electric shock, or burning and is limited to the area innervated by one or more branches of the trigeminal nerve. Each episode of pain is followed by a refractory period that can last from a few seconds to several minutes [4]. A prominent symptom is allodynia, in which a mere touch of the skin even by light clothing causes intense pain, in keeping with a process pointing at central sensitization and dysfunction of centrally situated second-order nociceptive neurons. Pain is refractory and may be very severe, leading to a considerable impairment of quality of life (QOL) and mental health in affected patients. Pain attacks usually occur spontaneously or by stimulating triggers, such as brushing teeth or shaving, usually located in the territory innervated by the trigeminal nerve [3,4,5,6]. 

Based on the etiology, TGN is classified into idiopathic, classic, and secondary TGN. The first is characterized by unknown causes and usually remains without a definite etiologic diagnosis. Classic TGN is associated with neurovascular compression (NVC) most frequently by the superior cerebellar artery of the trigeminal nerve roots into the pons. This compression usually results in the demyelination of nerve fibers, which then start firing ectopically. Secondary TGN may be caused by an underlying disease such as tumors or artery malformations and has also been associated with multiple sclerosis [1,7,8]. Generally, pain is propagated through the trigeminal pathway to the somatosensory cortex and the limbic system (central sensitization), while voltage-gated sodium channels (VGSCs) play a crucial role in the generation of ectopic activity in trigeminal afferents [9].

Carbamazepine (CBZ) or oxcarbazepine (OXC) are the medications (both are potent VGSC blockers) of choice for providing long-term effective relief but often are not efficacious or poorly tolerated. Lamotrigine, gabapentin, BoNTA, pregabalin, baclofen, and phenytoin can be used alone or in combination with other medications. It is suggested that surgery may be offered if the pain is not adequately controlled with pharmacological options or if these medications are not tolerated well. In individuals with classic TN, microvascular decompression is indicated as first-line surgery [10]. If no NVC is evident in neuroimaging, neuroablative treatment may be an option. Secondary TGN patients should be treated using the same principles as primary patients. It is advised that patients should receive psychosocial and nursing support in addition to medical and surgical care [11].

In terms of OnabotulinumtoxinA (BoNTA), its licensed use was only granted as a preventative treatment for chronic migraine in adults, based on the favourable safety/efficacy outcomes of the pooled analysis of PREEMPT trials [12]. Nonetheless, there is limited clinical evidence on its use in TGN; although tellingly, according to the results of the few available (n = 4) relevant double-blind randomized controlled trials, BoNTA may have a significant beneficial effect as an adjunct therapy in up to 70% of treatment-refractory patients with TGN when given as monotherapy or add-on to anticonvulsants [13]. Nonetheless, according to the latest European Academy of Neurology guidelines on TGN therapies, BoNTA was recommended as an add-on therapy for the medium-term treatment of TGN based on very low-quality evidence resulting from the study of small sample sizes and use of variable techniques and dosages, as also varying quality points deducted for risk of bias [14]. While some studies have investigated the efficacy and safety of BoNTA injections for the treatment of TGN, there are few available data on clinical outcomes in real-world conditions. Hence, real-world evidence generated even from case series can shed further light on the efficacy and safety of BoNTA injections for patients suffering from refractory TGN.

The purpose of this research was to report on the treatment’s clinical outcomes, such as improvements in pain scores, medication use, and quality of life, in a case series of patients with refractory TGN who had BoNTA injections as part of their treatment strategy. More specifically, we present here some additional data from a retrospective study that sought to assess the efficacy of combining pharmacological therapy with BoNTA add-on to CBZ or OXC in treatment-refractory patients with TN who failed to respond (less than 30% response rate) to adequate monotherapy.

## 2. Results

We reviewed the medical files of 15 treatment-refractory patients with TGN who attained combined treatment with a single BoNTA course add-on to CBZ or OXC. The follow-up period for each patient was 6 months. CBZ or OXC was delivered at median doses of 1200 mg (range: 800–1600) and 1800 mg (range: 1200–2400), respectively, at the time of evaluation for further treatment with BoNTA. The study sample consisted of 5 males (33.3%) and 10 females (66.7%) with a mean age of 58.5 ± 12.5 (range: 27–88) years. The distribution of TGN was as follows: five patients had V2 involvement in the maxillary division of the trigeminal nerve, three had V3 involvement, four had both V2 and V3, one had V1 involvement, and two had V1, V2, and V3 involvement. The mean duration of TGN was 25.9 ± 19.6 months (range 6–60). Eight patients had involvement of right side and seven of left side. The frequency of attacks was more than 5/day for all patients (range 5–>40 per day). All patients had failed to respond to at least two types of conventional treatments. Approximately all patients have tried paracetamol, NSAIDS, and tramadol when needed. Six of them have been on duloxetine as well, and fourteen have tried pregabalin in the past. Lidocaine patch has been tried by five patients. The patients underwent magnetic resonance imaging, which did not detect any structural pathology or suggest the need for surgical procedures. Table 1 describes in detail the baseline epidemiological and clinical characteristics of participants.

**Figure 1:**
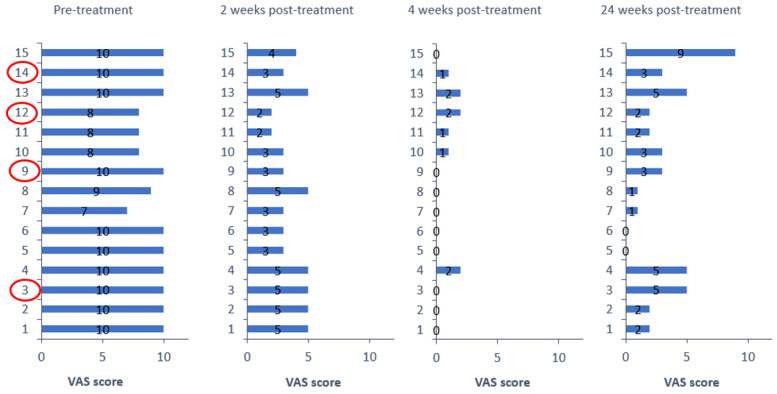
Effect of BoNTA on clinical outcomes. VAS score for every patient at pre-treatment and at 2 weeks, 4 weeks, and 24 weeks post-treatment, respectively.

All patients (15/15; 100%) reported a significant reduction in the severity of their neuropathic pain, which was related to their TGN, after the application of a single BoNTA session. The mean pain score on the VAS scale significantly decreased from 9.3 ± 1.1 to 3.7 ± 1.2 at 2 weeks after injecting BoNTA (*p* < 0.001); it decreased even more at 4 weeks after treatment, 0.6 ± 0.8; and it remained at significantly reduced levels 6 months after treatment, 2.9 ± 1.1 (*p* < 0.001). The mean duration of pain relief was 22.5 weeks (range 8–24). There was no significant difference in pain reduction between patients with maxillary versus mandibular involvement, while the same applied in patients with primary TGN at 2 weeks after injecting BoNTA, compared to those with secondary TGN. Likewise, the duration of clinical benefit was comparable in each TGN subgroup (primary vs. secondary). The overall clinical effect of BoNTA against TGN-related neuropathic pain is summarized in Figure 1. Patients in red cycle (3, 9, 12, 14) were those with secondary TGN, while patient 15 was the only case (primary TGN) who received the maximum delivered dose of 200 units (40 points; 5 units/0.1 mL per point).

Regarding the impact of BoNTA therapy on patients’ health-related quality of life (HRQOL), the mean sum score on the Short Form 36 (SF-36) questionnaire improved from 36.5 at baseline to 58.2 at 3 months after treatment. There were significant improvements in both the physical and mental health domains (*p* < 0.05) of SF-36 to further document the merits of treating pharmaco-refractory TGN patients with BoNTA.

**Figure 2:**
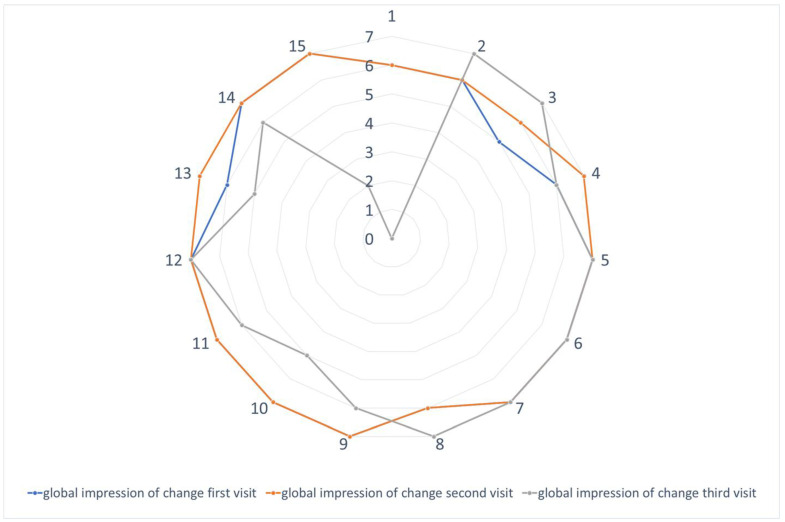
Effect of BoNTA on Patient Global Impression of Change (PGIC). PGIC score for every patient 2 weeks, 4 weeks, and 24 weeks post-treatment, respectively.

In terms of patients’ satisfaction, all patients reported being satisfied with the treatment to score ≥5 on “Patient Global Impression of Change” (PGIC) 2 weeks after treatment. Specifically, one patient scored 5, five patients scored 6, and nine patients scored 7 on PGIC. Notably, 10 patients (67%) reported that they would choose BoNTA injections as their first-line treatment option if they were to experience a TGN recurrence. The overall PGIC for every visit post-treatment is summarized in Figure 2. 

No major adverse events were observed in any of our enrolled patients. Mild and transient tenderness and/or erythema or wheals in the injection sites was reported by five patients, which resolved within 48 h. There was no evidence of facial asymmetry, masticatory abnormalities or other commonly reported significant complaints after BoNTA treatment for TGN. None of the patients required additional medications or procedures for pain relief during the study period. A total of 53% of patients stopped taking CBZ or OXC during the observational period without any relapses in their TGN-related pain. 

## 3. Discussion

The objective of our current research was to test the efficacy of BoNTA add-on to CBZ or OXC in treatment-refractory patients with TGN so as to add to the expanding body of literature on the topic and to provide physicians with useful insights into the possible role of this medication to interfere with the pathogenesis of TGN, in order to eventually achieve successful management of this quite difficult paradigm of neuropathic pain.

There is evidence to suggest that both peripheral and central sensitization and the involvement of the trigemino-vascular system form the major components of TGN pathogenesis [15]. Peripheral sensitization, defined as the reduced threshold for stimulation of the peripheral neurons, may be due to the chemical irritation of trigeminal fibers by inflammatory mediators. At the periphery, CGRP, glutamate, and other neurotransmitters released from sensory nerve endings lead to sensitization of meningeal trigeminal sensory afferents. On the other hand, central sensitization, characterized by abnormal activity of second-order trigeminal sensory neurons, including spontaneous firing, increased firing in response to painful stimulation, and a reduced threshold for firing in response to non-noxious stimuli, in keeping with allodynia, results as a consequence of increased incoming stimulation from the trigeminal nerve (peripheral sensitization) [16].

Natural or manufactured BoNTA is one of the most potent neurotoxins. It inhibits the presynaptic release of axonal acetylcholine (Ach) to limit neuromuscular transmission and to eventually cause muscle relaxation. In 2002, Micheli et al. were the first to identify BoNTA as a potential pharmacological option for providing clinically meaningful analgesia in TGN [17]. Further research has also demonstrated the efficacy of BoNTA in the treatment of various pain models, including TGN [18]. Indeed, with our results, we also provide evidence to support that BoNTA may be an excellent treatment option for refractory TGN without needing to cope with the poor safety/tolerability of conventional medications or risk of surgical interventions.

The actual mechanism of BoNTA action in providing pain reduction appears to be multifactorial and not yet fully elucidated. When BoNTA is injected directly into contracting muscles, it attaches to presynaptic nerve terminals and becomes internalized, preventing the neurotransmitter Ach from being exocytosed at neuromuscular junctions. BoNTA may also induce peripheral neurovascular activity by blocking neurotransmitter release, including substance P, neurokinin A, CGRP, noradrenaline, dopamine, enkephalin, and enteral polypeptide. These transmitters operate on blood vessels and glutamate, decreasing afferent nerve impulses and suppressing neurogenic inflammation in order to eventually inhibit peripheral sensitization by diminishing the number of pain signals that reach the brain and, indirectly, preventing central sensitization [19,20,21]. Changes in CGRP plasma levels in patients with classical TGN have been noted in several studies, and plasma CGRP concentrations were used to predict the response to BoNTA. More specifically, CGRP levels decrease significantly in patients with classical TGN after treatment with BoNTA, thoroughly suggesting that the analgesic mechanism of BoNTA may be indeed related to the inhibition of CGRP release [22]. Moreover, BoNTA has an antinociceptive effect, directly modifying central sensitization by suppressing excessive TRPA1, TRPV1, and TRPV2 expression in the spinal trigeminal nucleus. A recent study found that BoNTA’s antinociceptive effects are imparted via blocking Nav1.7 upregulation in the trigeminal ganglion. As a result, such actions are not limited to the neuromuscular junction but also act on central nervous system areas, including the trigeminal ganglion, trigeminal nerve ridge nucleus, or spinal cord [12,23]. Further research is warranted to shed additional light on the mechanisms through which BoNTA interferes with TGN pathogenesis and pain relief.

Four double-blind randomized controlled trials found that BoNTA injections were beneficial for patients with TGN, with considerable clinical benefits [24,25,26,27]. In most studies, roughly 70–90% of patients responded to BoNTA injections, and the mean pain severity and frequency were reduced by approximately 50–90% four weeks post-treatment. BoNTA was administered subcutaneously, intradermally, or submucosally in these investigations. Edema, hematoma, discomfort, facial asymmetry, and masticatory abnormalities were reported as being the most common side effects. The last two problems are associated with their effects on the masseter muscle. Furthermore, a recent systematic review and meta-analysis evaluated the efficacy and safety of BoNTA in patients with idiopathic TGN [28]. The results showed that BoNTA proved effective in significantly reducing pain intensity and improving the QOL in these patients The authors concluded that BoNTA may be a safe and effective treatment option for idiopathic TN, but due to the limited sample size and heterogeneity, further larger and more well-designed RCTs are imperative to validate their findings [28].

Furthermore, several case reports were published describing the effect of BoNTA administered directly to trigeminal ganglion or intradermally and/or submucosally in refractory TGN. New techniques in several cases have also been described, such as injecting BoNTA intraorally into the mental foramen, which resulted in pain relief as well [29,30]. Finally, the effectiveness of BoNTA has also been studied once in secondary TGN and more specifically in 53 patients with multiple sclerosis-related TGN. The treatment with subcutaneous injections to the painful area was found to be effective in at least 50% of treated patients [9]. 

The results we herein report further provide evidence to suggest that a single BoNTA course may be an effective treatment for treatment-refractory TGN patients, with 60.2% and 93.5% reduction in mean pain VAS scores at 2 weeks (9.3 to 3.7) and 4 weeks (9.3 to 0.6) after injecting BoNTA, respectively. The reduction of 60.2% in pain VAS at 2 weeks post-treatment in our study is similar to that previously systematically reviewed and reported (68% reduction in mean pain VAS from 7.5 before treatment to 2.4 post-treatment) [28]. Nonetheless, with our study we add some new findings to the available body of related literature by demonstrating that the beneficial effect of a single BoNTA course can last for up to 6 months, as was evidenced by significantly reduced mean pain VAS at 24 weeks after treatment, compared to baseline (2.9 from 9.3; 68.8% reduction). The same long-term beneficial effect was also demonstrated in a previous study which followed TGN patients for up to 14 months after BoNTA injections [31]. As such, subcutaneous BoNTA might be able to provide persistent clinically meaningful analgesia and significant improvement of patients’ QOL without any relapses in their TGN-related pain, while about half of the patients (53% in our study) stopped taking CBZ or OXC during this period. As such, BoNTA might be a suitable symptomatic, rather than prophylactic, treatment for refractory TGN patients. 

Generally, our findings are consistent with previous studies that have also demonstrated the efficacy of BoNTA in reducing pain associated with various chronic neuropathic pain conditions, including migraine headaches and chronic low back pain [32]. Tellingly, the use of BoNTA in the treatment of TGN has several advantages over other available treatment options, such as surgical interventions and medication. BoNTA injections are relatively safe and well-tolerated, with minimal side effects, as has been also documented in our study. In addition, BoNTA injections are minimally invasive and can be performed in an outpatient setting, allowing for convenient and easily accessible treatment.

Nonetheless, our study has some limitations that should be considered. Firstly, this was a retrospective, single-arm, uncontrolled case series. These limitations prevent us from making robust causal inferences about the effect of BoNTA on TGN. Secondly, the duration of the effect of BoNTA on TN is unclear, and further long-term research is needed to determine the optimal dosing and timing of injections. In addition, our sample size was relatively small, and a larger sample size is needed to confirm these findings. The national reimbursement policy that is currently applied expensive therapies, including BoNTA, restricted us from having a larger sample size, as BoNTA can only be approved to be fully reimbursed by the Greek National Health System as an off-label treatment option in TGN patients who are failing to respond to or have been unable to tolerate at least three previous first-line oral preventatives. As such, only a few applications pass through the Electronic Prior Authorization System (ePAS) to receive approval for commencement in patients in the context of full reimbursement. 

## 4. Conclusions

In conclusion, BoNTA injections may be a safe and effective treatment option for patients with refractory TGN when added-on to CBZ or OXC. The beneficial effect may last for 6 months after commencing a single BoNTA course; however, we should note that the persistence of the clinical benefit with BoNTA use at the follow-up of 24 weeks post-treatment might not be completely true, as TGN often has a remission period lasting from weeks to years where patients are pain-free [6]. However, the vast majority of our patients (14/15) remained at persistent remission 24 weeks post-treatment with VAS ≤5, and this long-term beneficial effect seems unlikely to be random and not directly attributed to BoNTA. Considering that the effectiveness of BoNTA may be dependent on the type of TGN (primary vs. secondary) and the dosage used, its use for TGN treatment may provide an alternative to surgical interventions and oral medications, providing patients with a minimally invasive, effective, safe, and well-tolerated treatment option. Nonetheless, considering that we report upon a retrospective, single-arm, uncontrolled case series, further well-designed, prospective placebo-controlled studies are warranted to determine the optimal dosing and timing of BoNTA injections so as to shed additional light on this clinically important issue concerning the adequate management of treatment-refractory TGN. 

## 5. Materials and Methods

After obtaining written, informed consent from each patient and approval from the institutional review board and ethics committee of the University Hospital of Larissa, Greece, in line with the principles of the Helsinki Declaration, we retrospectively reviewed the medical files of adult (>18 years) patients with a definite diagnosis of TGN, according to the criteria of the International Classification of Headaches Disorders-III [33]. Enrolled patients had to be previously treated with a single course of BoNTA injections as part of their treatment plan. In addition, patients were included in this retrospective study if they: (i) were considered as non-responders to at least 2 preventive oral medications, including CBZ or OXC, because of inefficacy (less than 30% reduction in pain severity and frequency of attacks) or intolerance; (ii) had agreed to keep pain diaries and patient-reported outcome scales; and (iii) were consistent in providing safety/tolerability data through monthly phone interviews. We excluded patients with comorbid diseases that may be exacerbated by BoNTA (e.g., myasthenia gravis, motor neuron disease, or Lambert–Eaton syndrome) or those who were receiving drugs with neuromuscular junction toxicity 10 days before BoNTA treatment, including quinine, aminoglycosides, or penicillamine. Patients with an infection of the skin or mucosa at any of the injection sites and those with a major psychiatric disorder or malignancy were also excluded. Finally, pregnant or nursing females as well as patients who previously participated in any investigational drug or device study within 6 months were not allowed to participate.

**Figure 3:**
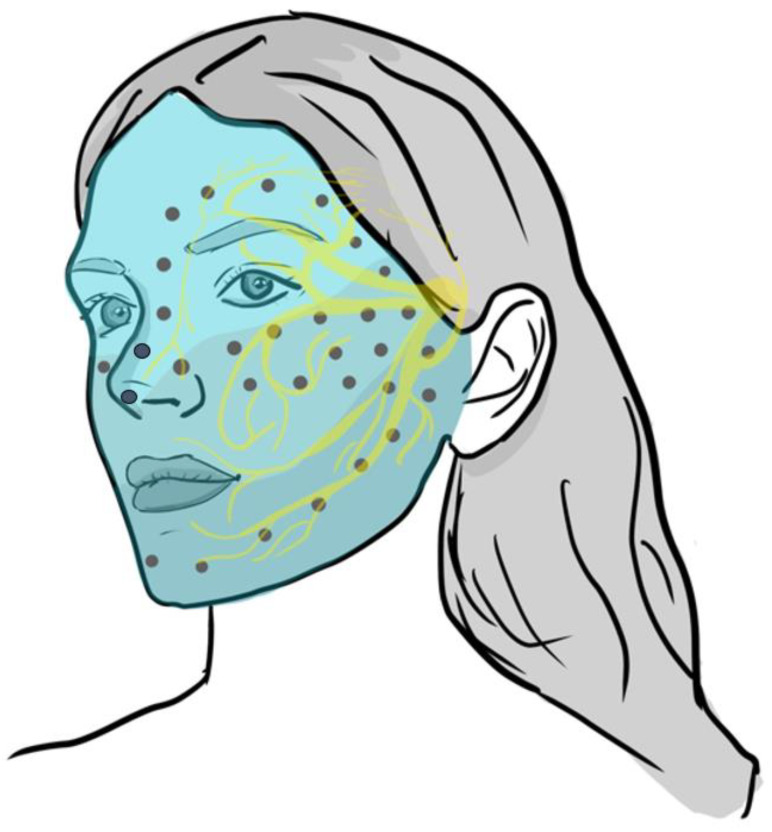
General template for applying BoNTA in painful areas of enrolled patients with TGN, according to the 3 divisions of the trigeminal nerve: (i) ophthalmic nerve comprised of frontal, lacrimal, and nasociliary branches, (ii) maxillary, and (iii) mandibular nerves.

BoNTA (Botox^®^ 100UI/fl; Allergan-Abbvie, Greece) was reconstituted with isotonic sodium chloride solution to reach a concentration of 5 or 2.5 units/0.1 mL. Administration was guided by each patient’s perceived pain and trigger zones, delivering BoNTA via a syringe of 1 mL. As such, subcutaneous injections of 5 units were administered at 1 cm intervals to the skin area where the patient described the pain, starting from the area where the pain was most intense. Subcutaneous BoNTA injections of 2.5 units/0.1 mL were selectively applied in areas with less intense pain. In this way, BoNTA was applied to the entire painful area as depicted in Figure 3. BoNTA injections were performed in all patients by the same physician (GX). The total dosages delivered varied, ranging from 30 to 200 units (mean ± standard deviation: 87.3 ± 39.2) at up to 40 injections points (5 units/0.1 mL in just one patient [case 15 in Figure 1] who received the maximum dose of 200 units in a single BoNTA course due to significant affectation of all 3 trigeminal nerve branches). In any case, BoNTA was applied at a median number of 20 points (range: 6–40), mostly commencing at 5 rather than 2.5 units/0.1 mL per point, according to every patient’s needs. 

As part of our practice, all patients had to complete ratings at specific interviews during the course of standard care, and the corresponding data were recorded in their medical files. As such, we retrospectively collected data on patients’ demographics, clinical characteristics of TGN, previous treatments and efficacy outcomes, details of the BoNTA injection schemes, and their corresponding clinical outcomes. Clinical outcomes included changes in pain scores (as measured by a visual analog scale (VAS) or numerical rating scale from 0–10, with higher score to indicate worse severity) and duration of TGN remission. We generally considered patients with an at least a 50% reduction in VAS score and/or pain frequency at 2 weeks post-BoNTA, compared to pre-treatment, as responders. Furthermore, we also assessed for every patient the consistency (duration) of BoNTA clinical effect with the use of a VAS score at 4 weeks and 24 weeks post-treatment. Notably, the 24-week results were obtained after delivering just a single course of BoNTA, as previously described, and not after a second repeated BoNTA session at 12 weeks.

Secondary outcomes included the changes in HRQOL, as measured by a validated questionnaire such as the SF-36, which is also available and validated in Greek language [34,35]. Briefly, SF-36 is widely used to disclose the patients’ reported perceptions of their own health and well-being and includes eight scales to eventually yield physical (physical functioning 10 items; role-physical 4 items; bodily pain 2 items; and general health 5 items) as well as mental health compound (summary) scores. The mental health subscore is composed of vitality: 4 items; social functioning: 2 items; role–emotional: 3 items; and mental health: 5 items. SF-36 scoring has a range from 0 to 100, with higher scores being indicative of better health status [34]. 

The satisfaction of patients to BoNTA was also recorded with a short self-report 7-point PRO questionnaire “Patient Global Impression of Change” (PGIC) (1 stands for “no change” and 7 for “considerable improvement”). Patients with PGIC 5 had “clinically significant benefit”, whereas patients with PGIC 4 had “no benefit [36].

### Statistical Analysis

Descriptive statistics were computed depending on the nature of the analyzed variable, including means, standard deviations, and percentages. The changes in subsequent pain scores and overall clinical efficacy scores from baseline vs. post-BoNTA were assessed using paired samples *t*-tests. The same approach was followed to assess all secondary outcomes. Unless otherwise stated, all tests were two-sided, and significance was set at *p* < 0.05. The SPSS for Windows (release 27.0; SPSS Inc., Chicago, IL, USA) performed the statistics. 

## Figures and Tables

**Table 1:** Demographic and clinical characteristics of participants at baseline.

*Participants n* = 15		
*Variable*	N	%
**Gender**		
Females	10	66.7
Males	5	33.3
**Age ± SD (range)**	55.8 ± 12.5 (27–88)	
**Type of TGN**		
Primary	11	
secondary	4	
**Trigeminal nerve branch involved**		
**V1**	1	
**V2**	5	
**V3**	3	
**V2 + V3**	4	
**V1 + V2 + V3**	2	
**Months ± SD (range) with TGN**	25.9 ± 19.6 (6–60)	

## Data Availability

The data that support the findings of this study are available from the corresponding author upon reasonable request.
